# Latent class analysis of chronic disease co-occurrence, clustering and their determinants in India using Study on global AGEing and adult health (SAGE) India Wave-2

**DOI:** 10.7189/jogh.14.04079

**Published:** 2024-06-28

**Authors:** Neha Shri, Saurabh Singh, Shri Kant Singh

**Affiliations:** Department of Survey Research and Data Analytics, International Institute for Population Sciences, Mumbai, India

## Abstract

**Background:**

Understanding chronic disease prevalence, patterns, and co-occurrence is pivotal for effective health care planning and disease prevention strategies. In this paper, we aimed to identify the clustering of major non-communicable diseases among Indian adults aged ≥50 years based on their self-reported diagnosed non-communicable disease status and to find the risk factors that heighten the risk of developing the identified disease clusters.

**Methods:**

We utilised data from the nationally representative survey Study on Global AGEing and Adult Health (SAGE Wave-2). The eligible sample size was 6298 adults aged ≥50 years. We conducted the latent class analysis to uncover latent subgroups of multimorbidity and the multinomial logistic regression to identify the factors linked to observed latent class membership.

**Results:**

The latent class analysis grouped our sample of men and women >49 years old into three groups – mild multimorbidity risk (41%), moderate multimorbidity risk (30%), and severe multimorbidity risk (29%). In the mild multimorbidity risk group, the most prevalent diseases were asthma and arthritis, and the major prevalent disease in the moderate multimorbidity risk group was low near/distance vision, followed by depression, asthma, and lung disease. Angina, diabetes, hypertension, and stroke were the major diseases in the severe multimorbidity risk category. Individuals with higher ages had an 18% and 15% higher risk of having moderate multimorbidity and severe multimorbidity compared to those in the mild multimorbidity category. Females were more likely to have a moderate risk (3.36 times) and 2.82 times more likely to have severe multimorbidity risk.

**Conclusions:**

The clustering of diseases highlights the importance of integrated disease management in primary care settings and improving the health care system to accommodate the individual’s needs. Implementing preventive measures and tailored interventions, strengthening the health and wellness centres, and delivering comprehensive primary health care services for secondary and tertiary level hospitalisation may cater to the needs of multimorbid patients.

Chronic diseases have emerged as a significant global health challenge, substantially impacting both developed and developing nations. According to the World Health Organization (WHO), chronic diseases account for approximately 74% of all global deaths, with 77% occurring in low- and middle-income countries like India [1]. These diseases, also known as non-communicable diseases (NCDs), encompass various conditions, including cardiovascular diseases, diabetes, chronic respiratory diseases, and cancers, contributing to a substantial portion of global morbidity, mortality, and health care costs [2]. Over the past few decades, the global disease burden has shifted from communicable to NCDs, reflecting demographic and epidemiological transitions [3]. The shift is primarily attributed to rapid urbanisation, sedentary lifestyles, unhealthy dietary practices, and the ageing of populations [4].

Chronic diseases often exhibit complex interrelationships. Evidence suggests that individuals with one chronic disease are more susceptible to developing other chronic conditions [5]. The co-occurrence or clustering of multiple chronic conditions within an individual, known as multimorbidity, is gaining attention due to its adverse impact on health outcomes, health care costs, and quality of life. The simultaneous presence of multiple chronic conditions can exacerbate each other effects, leading to worse health outcomes and higher health care costs [6]. The clustering of chronic diseases poses challenges in resource allocation, health service delivery, and policy formulation [7]. Furthermore, research has shown the intriguing concept of disease clustering, wherein certain chronic diseases tend to co-occur more frequently than expected by chance alone. For instance, cardiovascular diseases and diabetes often cluster, sharing common risk factors such as obesity and hypertension [8].

Multimorbidity is not solely an issue of ageing populations; it affects individuals across different age groups [6], with a significant impact on older adults [9]. NCDs disproportionately affect people in low- and middle-income countries, where more than three-quarters of global NCD deaths (31.4 million) occur [1]. Research has highlighted the existence of health inequalities, with chronic diseases disproportionately affecting vulnerable and marginalised communities [10]. As populations age and societies undergo epidemiological transitions, the burden of these diseases is anticipated to rise. According to estimates, 4.7 million people in India died in 2017 from NCDs, accounting for 49% of all causes of death [11], reflecting the significant health challenges posed by chronic diseases.

India stands at the intersection of an epidemiological transition where the burden of chronic diseases increases alongside persistent challenges related to infectious diseases and maternal and child health [12]. Reports suggest that cardiovascular diseases are the leading cause of mortality in India (27%), followed by respiratory diseases (9%), cancer (6%), and diabetes (2.4%) [11]. Recent studies have highlighted the prevalence of multimorbidity globally, underscoring the need for comprehensive approaches to disease management and health care planning [4]. In India, where communicable diseases have historically dominated the public health agenda, the increasing prevalence of chronic diseases presents a dual burden. The co-occurrence of conditions like diabetes and cardiovascular diseases in India has been widely documented [13]. Additionally, the prevalence of clustering of multiple risk factors associated with chronic NCDs is substantially high in India and has increased between 2005–06 and 2015–16 [14].

Understanding chronic disease prevalence, patterns, and co-occurrence is important for effective health care planning and disease prevention strategies. There is a need to explore how chronic diseases cluster across populations, geographical regions, and socio-economic strata [15]. Recognising that the impact of multiple lifestyle risk factors can be more detrimental to health than individual factors, in this research we focus on determinants, i.e. socio-demographic and behavioural factors linked to the multimorbidity latent classes. We shed light on the intricate interplay of these ailments in India and underscore the urgency of addressing their co-occurrence and clustering. The objective of the paper was to find out the clustering of major NCDs among Indian adults aged ≥50 years based on their risk for multimorbidity, utilising variables of self-reported diagnosed NCDs through latent class analysis. Additionally, we aimed to identify to what extent socio-demographic, anthropometric and behavioural factors impact the clustering of NCDs among the Indian adult population. This investigation will fill a critical gap in understanding the multi-dimensional risk factor landscape of NCD clusters in India.

The novelty of this study lies in its targeted investigation of the clustering of major NCDs among Indian adults aged ≥50 years, a focus not extensively explored in previous Indian research [16,17]. While similar studies have been conducted in other nations [18–20], this research fills a significant gap in the Indian context. Prior studies in India have primarily concentrated on the determinants and prevalence of NCDs or the clustering of risk factors [14,21], not directly on the diseases’ clustering. By utilising latent class analysis for self-reported diagnosed NCDs, we offer a new perspective on multimorbidity patterns in India, examining how socio-demographic, anthropometric, and behavioural factors influence these clusters. This approach promises to enhance the understanding of NCD clusters in India, providing valuable insights for effective health care planning and disease prevention.

## METHODS

### Data

The secondary data for this study were drawn from the second wave of the Study on Global AGEing and Adult Health (SAGE Wave-2, India, 2015) supported by the WHO. WHO’s SAGE is a longitudinal study collecting data on adults aged ≥50 years, plus a smaller comparison sample of adults aged 18–49 years, from nationally representative samples in China, Ghana, India, Mexico, the Russian Federation, and South Africa. It is also supported by the United States National Institute on Ageing, the Division of Behavioural and Social Research, and national governments. SAGE is a comprehensive survey that assesses various aspects of health and well-being among older adults. The data set encompasses various socio-demographic, lifestyle, and health-related variables. In India, the data are collected from six states – Assam, Karnataka, Maharashtra, Rajasthan, Uttar Pradesh, and West Bengal. The SAGE India Wave-2 survey conducted in 2015 covered 9116 individuals from 8152 households [22]. Individuals aged ≥50 years were only considered in this study sample. After excluding the samples with missing information, the eligible sample size for the analysis was 6298 individuals.

### Variables

This analysis included nine chronic health conditions, namely angina pectoris, arthritis, asthma, chronic lung disease, diabetes mellitus, hypertension, stroke, visual impairment, and depression. All of these conditions (except visual impairment) were assessed through a question: ‘Have you ever been told by a health professional or doctor that you have (disease name)?’ The Computer-Assisted Personal Interviews enabled vision tests measuring near and distance vision for both eyes. Near vision was measured using a prescribed distance of 40 cm, and distance vision was measured at four meters. The respondents were classified as having multimorbidity if they had two or more morbid conditions simultaneously.

The variables employed in this study included age, sex, marital status, education, place of residence, adding salt at the dining table, individuals’ consumption of tobacco and alcohol, working status, physical activity, and self-rated health. SAGE incorporated a separate health examination and biomarkers module, including measures of anthropometry that measured weight, height, waist, and hip circumferences. The body mass index (BMI) values for individuals were categorised as underweight if their BMI was <18.5, normal if their BMI was 18.5–24.9, and overweight or obese if their BMI was ≥25.0.

The information on the wealth quintile was grouped into five categories – poorest, poorer, middle, richer, and richest. The respondent’s caste was categorised into scheduled tribes, scheduled castes, others, and other backward and their religion was distinguished as Hindu, Islam, and others. A wealth index was derived from household ownership of durable goods, dwelling characteristics (type of floors, walls, and cooking stoves), and access to improved water, sanitation, and cooking fuel [23]. Using a Bayesian post-estimation (empirical Bayes) method, households were arranged on the asset ladder, where the raw continuous income estimates were transformed in the final step into quintiles [22].

### Statistical methods

The bivariate analysis explored associations between pairs of chronic diseases and demographic variables. To uncover latent subgroups of individuals with similar patterns of chronic disease occurrences among the eligible participants, the latent class analysis was carried out in STATA, version 16 (StataCorp LLC, College Station, Texas, USA). Angina, arthritis, chronic lung disease, near/distant vision impairment, asthma, stroke, depression, diabetes, and hypertension were included as observable markers in the current investigation. The Bayesian information criterion, proven to offer reliable indications of class enumeration with categorical outcomes, was used to identify the ideal number of latent classes [24]. The lowest values of the Bayesian information criterion showed the model that best fits the data when comparing several feasible class models.

Once the best model was chosen, each participant was classified into one class based on the most significant calculated membership probability. The optimal number of latent classes was determined using the Bayesian information criterion, and three latent classes were considered in this study. The three latent classes were termed mild multimorbidity, moderate multimorbidity, and severe multimorbidity risk (based on the probabilities of having each of nine chronic conditions with low probability to moderate and severe probability) (Table S1 in the **Online Supplementary Document**).

Multinomial logistic regression was performed to identify the sociodemographic, anthropometric, and behavioural variables linked to observed latent class membership. The current work used STATA terminology to refer to multinomial logistic regression. Relative risk ratios (RRRs) with 95% confidence intervals (CIs) and corresponding *P*-values are presented for each explanatory variable.

## RESULTS

Most participants were aged 50–59 years (43%) and 60–69 years (37%). The study population was almost evenly divided between males and females. Most participants reported residing in rural areas (78%), cohabiting (77%). Moreover, 47% reported not having any formal education. Further, 66% never smoked, and 88% never consumed alcohol. More than two-thirds of the participants (69%) reported ever working, and 56% were physically active. The distribution of BMI indicated that 20% of participants were underweight, 56% had a normal BMI, and 24% were categorised as overweight or obese. The distribution of self-rated health showed that 37% of participants rated their health as good and 49% as moderate. Among participants, 72% reported good sleep quality, while 28% reported poor sleep quality. Participants were distributed across various states, with Uttar Pradesh having the highest representation (21%) and Assam having the least (10%) (**Table 1**).

**Table 1:** Participants characteristics, SAGE Wave-2, 2015 (n = 6298)

Characteristics	N (%)
Age in years	
*50–59*	2620 (42.95)
*60–69*	2318 (36.67)
*70–79*	1107 (16.57)
*80+*	253 (3.81)
Sex	
*Male*	2954 (49.88)
*Female*	3344 (50.12)
Marital status	
*Cohabiting*	4752 (77.34)
*Not-cohabiting*	1546 (22.66)
Education	
*No formal education*	3139 (46.72)
*Less than primary*	1709 (27.56)
*Secondary school*	1102 (19.21)
*College and above*	348 (6.51)
BMI	
*Underweight*	1655 (20.20)
*Normal*	3461 (55.47)
*Overweight/obese*	1182 (24.33)
Salt available at the dining table	
*No*	1803 (28.71)
*Yes*	4495 (71.29)
Smoking	
*Never*	4158 (66.47)
*Ever*	2140 (33.53)
Alcohol consumption	
*Never*	5858 (88.3)
*Ever*	713 (11.7)
Ever worked	
*Yes*	4220 (68.51)
*No*	2078 (31.49)
Physical activity	
*Vigorous*	1036 (16.59)
*Moderate*	1619 (25.02)
*Light*	874 (14.13)
*No activity*	2769 (44.26)
Self-rated health	
*Good*	2216 (36.58)
*Moderate*	3073 (48.45)
*Bad*	1009 (14.97)
Sleep quality	
*Good*	4479 (71.9)
*Bad*	1819 (28.10)
Waist circumference, x̄ (SD)	85.17 (11.48)
Hip circumference, x̄ (SD)	90.88 (10.19)
Wealth quantiles	
*Poorest*	1195 (17.23)
*Poorer*	1155 (17.49)
*Middle*	1166 (17.86)
*Richer*	1303 (21.09)
*Richest*	1479 (26.34)
Place of residence	
*Rural*	5012 (77.89)
*Urban*	1286 (22.11)
Caste	
*Scheduled tribe*	471 (7.14)
*Scheduled caste*	1029 (15.29)
*Other*	1888 (30.95)
*OBC*	2910 (46.61)
Religion	
*Hinduism*	5277 (83.57)
*Islam*	779 (12.41)
*Other*	242 (4.02)
State	
*Assam*	661 (10.19)
*Karnataka*	693 (11.33)
*Maharashtra*	1059 (17.45)
*Rajasthan*	1295 (21.57)
*Uttar Pradesh*	1346 (20.72)
*West Bengal*	1244 (18.74)

BMI – body mass index, OBC – other backward castes, SD – standard deviation, x̄ – mean

The major prevalent morbidity in the sampled respondents is hypertension (24%), followed by arthritis (19%), diabetes (11%), asthma (5%), angina (4%), depression (3%), lung disease (2%) and stroke (2%). Around half of the respondents (52%) reported low near and distance vision. Forty-two per cent of participants reported one, 21% reported two, and 12% reported three or more morbidities. Among the participants, 33% had multiple co-morbidities, while the remaining 67% did not report having multiple co-morbidities (**Table 2**).

**Table 2:** Descriptive statistics of type of morbidity prevalent among the participants, SAGE Wave-2, 2015 (n = 6298)

Morbidity condition	N (%)
Angina	
*No*	6079 (96.25)
*Yes*	219 (3.75)
Arthritis	
*No*	5089 (80.58)
*Yes*	1209 (19.42)
Asthma	
*No*	5952 (94.63)
*Yes*	346 (5.37)
Diabetes	
*No*	5655 (88.63)
*Yes*	643 (11.37)
Hypertension	
*No*	4884 (76.15)
*Yes*	1414 (23.85)
Lung disease	
*No*	6150 (97.70)
*Yes*	148 (2.30)
Stroke	
*No*	6149 (97.53)
*Yes*	149 (2.47)
Vision	
*Normal*	2893 (47.72)
*Low near/distance vision*	3405 (52.28)
Depression	
*No*	6142 (97.49)
*Yes*	156 (2.51)
Number of morbidities	
*0*	1562 (25.14)
*1*	2739 (42.32)
*2*	1294 (20.91)
*3*	502 (8.26)
*4*	157 (2.57)
*5*	40 (0.73)
*6*	1 (0.01)
*7*	2 (0.03)
*8*	1 (0.01)
Multimorbidity	
*No*	4301 (67.46)
*Yes*	1997 (32.54)

**Figure 1:**
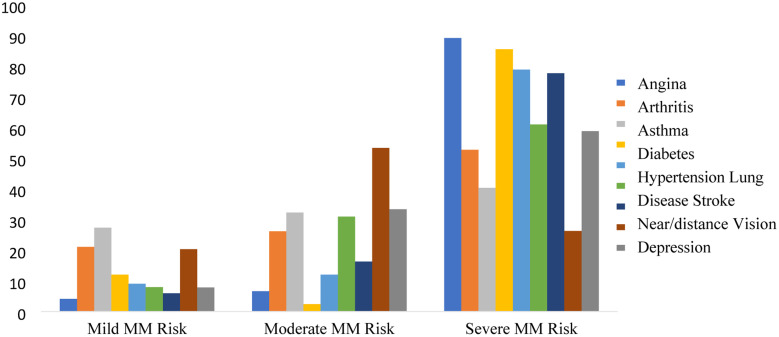
Latent class membership of morbidity condition, SAGE Wave-2, 2015.

Bivariate analysis explored the relationships between background characteristics and three latent classes (**Table 3**). Notably, respondents aged 50–59 were predominantly distributed in moderate multimorbidity (61.32%) and severe multimorbidity (22.55%), while those aged ≥80 and above were notably concentrated in moderate multimorbidity risk (57.49%) and severe multimorbidity risk (41%). Males were predominantly in mild multimorbidity (49.98%), while females showed a higher distribution in mild multimorbidity risk (35.99%) and mild multimorbidity (32.66%). Rural residents were more in the mild risk category, while respondents with college and above education and urban residents were notably present in the severe multimorbidity category (48.4% and 51.16%). BMI demonstrated significant clustering. Underweight individuals (59.12%) and those with bad sleep quality (41.21%) were primarily at moderate multimorbidity risk, whereas overweight and obese participants were notably concentrated at severe multimorbidity (53.1%). Respondents who had ever smoked and took alcohol were predominantly distributed in mild multimorbidity (41.91% and 43.72%, respectively). Individuals who worked (45.86%), had vigorous physical activity (50.08%), and had good self-rated health (62.22%) were primarily in mild multimorbidity. At the same time, those with bad self-rated health were notably concentrated in moderate multimorbidity risk (47.62%). Wealth quantiles exhibited distinctive clustering. The poorest (53.82%) and poorer (43.15%) wealth quantiles were predominantly found in moderate multimorbidity risk, while the wealthiest quantile was notably concentrated in severe multimorbidity (47.11%). In the mild multimorbidity risk group, the most prevalent diseases were asthma and arthritis, and the primary prevalent disease in the moderate risk group was low near/distance vision, followed by depression, asthma, and lung disease. Diseases such as angina, diabetes, hypertension, and stroke were the major diseases in the severe risk category (**Figure 1**, Figure S1 in the **Online Supplementary Document**).

**Table 3:** Characteristics of participants by latent class category, SAGE Wave-2, 2015 (n = 6298)

Characteristics	Moderate MM risk (%)	Mild MM risk (%)	Severe MM risk (%)	*P*-value
Age in years				<0.01
*50–59*	16.13	61.32	22.55	
*60–69*	33.51	34.82	31.67	
*70–79*	53.39	12.96	33.65	
*80+*	57.49	1.34	41.17	
Sex				<0.01
*Male*	24.48	49.98	25.53	
*Female*	35.99	32.66	31.34	
Marital status				<0.01
*Cohabiting*	25.43	48.02	26.55	
*Not-cohabiting*	46.72	18.37	34.91	
Education				<0.01
*No formal education*	45.29	34.87	19.84	
*Less than primary*	24.63	44.71	30.67	
*Secondary school*	10.88	49.72	39.41	
*College and above*	3.37	48.22	48.40	
BMI				<0.01
*Underweight*	59.12	32.71	8.17	
*Normal*	28.48	46.51	25.01	
*Overweight/obese*	10.32	36.58	53.10	
Salt available at the dining table				<0.01
*No*	33.78	34.79	31.44	
*Yes*	28.83	43.93	27.24	
Smoking				<0.01
*Never*	29.03	40.99	29.97	
*Ever*	32.67	41.91	25.42	
Alcohol consumption			40.98	<0.01
*Never*	31.23	40.98	27.78	
*Ever*	22.84	43.72	33.44	
Ever worked				<0.01
*Yes*	28.91	45.86	25.23	
*No*	33.17	31.40	35.43	
Physical activity				<0.01
*Vigorous*	27.48	50.08	22.44	
*Moderate*	34.43	33.65	31.92	
*Light*	28.09	44.53	27.38	
*No activity*	29.62	41.31	29.07	
Self-rated health				<0.01
*Good*	17.26	62.22	20.52	
*Moderate*	34.69	34.54	30.76	
*Bad*	47.62	12.06	40.33	
Sleep quality				<0.01
*Good*	25.97	49.27	24.76	
*Bad*	41.21	20.93	37.86	
Waist circumference in cm, x̄ (SD)	81.00 (10.15)	54.20 (10.38)	92.34 (11.50)	
Hip circumference in cm, x̄ (SD)	87.34 (9.03)	90.47 (9.20)	96.31 (10.84)	
Wealth quantiles				<0.01
*Poorest*	53.82	34.88	11.30	
*Poorer*	43.15	39.08	17.77	
*Middle*	34.10	43.02	22.89	
*Richer*	21.68	45.62	32.70	
*Richest*	10.53	42.36	47.11	
Place of residence				<0.01
*Rural*	34.96	43.04	22.00	
*Urban*	13.65	35.19	51.16	
Caste				<0.01
*Scheduled tribe*	33.11	52.55	14.34	
*Scheduled caste*	36.93	41.13	21.95	
*Other*	24.94	41.06	34.00	
*OBC*	31.15	39.80	29.05	
Religion				<0.01
*Hinduism*	30.47	42.12	27.42	
*Islam*	33.63	33.30	33.06	
*Other*	15.37	49.05	35.59	
State				<0.01
*Assam*	14.67	47.12	38.21	
*Karnataka*	25.48	35.20	39.33	
*Maharashtra*	25.55	47.15	27.30	
*Rajasthan*	25.29	48.46	26.25	
*Uttar Pradesh*	43.60	38.77	17.63	
*West Bengal*	36.94	30.95	32.11	
Total	30.25	41.30	28.45	

BMI – body mass index, MM – multimorbidity, OBC – other backward castes, SD – standard deviation, x̄ – mean

The results obtained from multinomial logistic regression were employed to understand the associations between background characteristics and the latent classes (**Table 4**). Age, sex, marital status, education, BMI, working, physical activity, self-rated health, sleep quality, waist circumference, wealth quintile, caste, and state were significantly associated with moderate and severe multimorbidity. However, alcohol use, smoking, and urban residence were significantly associated with an increased risk of having severe multimorbidity concerning individuals in the mild risk category. Individuals with higher ages were at increased risk of having moderate multimorbidity risk (RRR = 1.18; 95% CI = 1.16–1.19) and severe multimorbidity risk (RRR = 1.15 (95% CI = 1.13–1.16) than individuals from mild risk category. Females were 3.36 times more likely to have a moderate relative risk ratio (RRR = 3.36; 95% CI = 2.67–4.25) and 2.82 times more likely to have severe multimorbidity risk (RRR = 2.82; 95% CI = 2.19–3.61) than their male counterparts from mild risk category. Individuals with a college degree or above had 2.51 times more risk of belonging to the severe multimorbidity class and 89% less likely to belong to the moderate multimorbidity class relative to the mild multimorbidity risk group. Being obese or overweight was associated with a twice higher risk of being in the severe group. Bad self-rated health was associated with an eight times increased risk of being in the moderate risk group and a 16 times increased risk of being in the severe group. Individuals in the highest wealth quintile were at an increased risk of belonging to the severe multimorbidity class than those in the poorest wealth quintile. Residing in an urban area, smoking, alcohol consumption, not working ever, and individuals from other backward castes and other castes were associated with an increased likelihood of being in the severe group.

**Table 4:** Multinomial logistics regression analysis for factors associated with latent class analysis-based multimorbidity clusters as the exposure, SAGE Wave-2, 2015

Characteristics	Moderate MM risk, RR (95% CI)	Severe MM risk, RR (95% CI)
Age in years	1.18 (1.16–1.19)*	1.15 (1.13–1.16)*
Sex		
*Male*	ref.	ref.
*Female*	3.36 (2.67–4.25)*	2.82 (2.19–3.61)*
Marital status		
*Cohabiting*	ref.	ref.
*Not-cohabiting*	2.13 (1.74–2.62)*	2.31 (1.86–2.88)*
Education		
*No formal education*	ref.	ref.
*Less than primary*	0.59 (0.48–0.72)*	1.62 (1.30–2.01)*
*Secondary school*	0.28 (0.21–0.37)*	2.2 (1.71–2.84)*
*College and above*	0.11 (0.06–0.21)*	2.51 (1.72–3.67)*
BMI		
*Underweight*	ref.	ref.
*Normal*	0.41 (0.33–0.50)*	1.27 (0.97–1.66)‡
*Overweight/obese*	0.17 (0.12–0.25)*	1.89 (1.32–2.72)*
Salt available at the dining table		
*No*	ref.	ref.
*Yes*	0.88 (0.73–1.06)	0.77 (0.63–0.95)†
Smoking		
*Never*	ref.	ref.
*Ever*	0.96 (0.80–1.15)	1.34 (1.10–1.63)*
Alcohol consumption		
*Never*	ref.	ref.
*Ever*	1.21 (0.91–1.60)	2.24 (1.71–2.93)*
Ever worked out		
*No*	ref.	ref.
*Yes*	0.82 (0.66–1.00)*	1.73 (1.38–2.16)‡
Physical activity		
*Vigorous*	ref.	ref.
*Moderate*	1.11 (0.87–1.42)	0.99 (0.76–1.28)
*Light*	0.77 (0.58–1.03)‡	0.73 (0.54–0.99)†
*No activity*	0.74 (0.59–0.94)†	0.61 (0.48–0.79)*
Self-rated health		
*Good*	ref.	ref.
*Moderate*	2.98 (2.51–3.55)*	3.56 (2.96–4.28)*
*Bad*	7.61 (5.72–10.12)*	15.56 (11.49–21.08)*
Sleep quality		
*Good*	ref.	ref.
*Bad*	2.61 (2.18–3.14)*	3.06 (2.53–3.70)*
Waist circumference in cm	1.03 (1.02–1.05)*	1.08 (1.06–1.10)*
Hip circumference in cm	1.00 (0.98–1.02)	1.00 (0.98–1.01)
Wealth quantiles		
*Poorest*	ref.	ref.
*Poorer*	0.86 (0.68–1.09)	1.15 (0.83–1.60)
*Middle*	0.60 (0.47–0.77)*	1.20 (0.88–1.65)
*Richer*	0.38 (0.30–0.49)*	1.54 (1.13–2.09)*
*Richest*	0.26 (0.19–0.34)*	2.00 (1.46–2.73)*
Place of residence		
*Rural*	ref.	ref.
*Urban*	0.92 (0.72–1.17)	1.97 (1.61–2.40)*
Caste		
*Scheduled tribe*	ref.	ref.
*Scheduled caste*	1.29 (0.93–1.79)	1.99 (1.33–3.00)*
*Other*	1.42 (1.03–1.96)†	1.90 (1.30–2.79)*
*OBC*	1.71 (1.26–2.31)*	2.54 (1.74–3.70)*
Religion		
*Hinduism*	ref.	ref.
*Islam*	1.04 (0.81–1.35)	1.63 (1.26–2.11)*
*Other*	0.64 (0.40–1.02)‡	1.33 (0.90–1.97)
State		
*Assam*	ref.	ref.
*Karnataka*	3.18 (2.16–4.70)*	0.8 (0.56–1.13)
*Maharashtra*	2.55 (1.78–3.65)*	0.44 (0.32–0.61)*
*Rajasthan*	2.23 (1.58–3.15)*	0.50 (0.36–0.68)
*Uttar Pradesh*	6.75 (4.77–9.55)*	0.24 (0.17–0.34)
*West Bengal*	6.18 (4.34–8.79)*	0.55 (0.39–0.76)

BMI – body mass index, CI – confidence interval, MM – multimorbidity, OBC – other backward castes, ref – reference, RR – risk ratio

*Significant at 99% confidence level.

†Significant at 95% confidence level.

‡Significant at 90% confidence level.

## DISCUSSION

In this study, we showed that the prevalence of single morbidity was the highest (42%), while the coexistence of morbidity was prevalent in 32% of the sampled individuals. The latent class analysis grouped our sample of men and women aged ≥50 years into three groups – mild (41%), moderate (30%), and severe (29%) multimorbidity risk. In the mild multimorbidity risk group, the most prevalent diseases were asthma and arthritis, and the major prevalent disease in the moderate multimorbidity risk group was low near/distance vision, followed by depression, asthma, and lung disease. Diseases such as angina, diabetes, hypertension, and stroke were the major diseases in the severe multimorbidity risk category. These various NCDs in the study region could indicate that they share common risk factors, have a similar pattern of occurrence, or are linked causally [25]. Previous research that employed the latent class analysis technique to explain patterns of chronic illness coexistence in older populations has produced varied findings regarding the number of clusters found. For instance, two latent class clusters, namely low co-morbidity and hypertension-diabetes-arthritis were identified in a study conducted in India among individuals aged 15–64 years [16]. A study reported six clusters, i.e. relatively healthy, hypertension, gastrointestinal disorders-hypertension-musculoskeletal disorders, musculoskeletal disorders-hypertension-asthma, metabolic disorders, and complex cardiometabolic disorders among individuals aged ≥45 years, using data from the Longitudinal Ageing Study in India (2017–18) [17]. Similar to our findings, a study conducted in South Africa reported three groups – minimal multimorbidity risk, concordant (hypertension and diabetes), and discordant (angina, asthma, chronic lung disease, arthritis, and depression) [20]. A similar study conducted in Jamaica revealed four distinct profiles – a relatively healthy class (52.70%), with a single or no morbidity, and three multimorbid classes, i.e. metabolic (30.88%), vascular-inflammatory (12.21%), and respiratory (4.20%) [23]. Similar has also been observed in other parts of the world [25].

Very few studies have been conducted in India to understand the prevalence and predictors of multimorbidity using a probability-based classification of chronic disease coexistence [16,17]. However, it is challenging to compare these studies depending on the number and kind of diseases included in the study, the sample, and the methodology used to gather disease data. Previous studies have reported higher chances of individuals being in the hypertension-diabetes-arthritis group [16] and latent classes with a higher prevalence of diabetes and hypertension [17]. Compared with the mild multimorbidity risk group, an increase in age, being female, and not cohabitating were associated with belonging to the moderate multimorbidity group. Tobacco use and increase in age were associated with belonging to the severe multimorbidity group. In line with the previous findings, higher age was significantly associated with an increased relative risk of being at moderate or severe multimorbidity risk [17,18,20,25,26].

Limited income restricts access to nutritious food, safe housing, and quality health care, fuelling the rise of chronic conditions like diabetes, hypertension, and respiratory illnesses [27,28]. Lower education levels are associated with poorer health literacy, hindering understanding of preventive measures and early detection of diseases [29]. This can lead to the clustering of conditions as one disease progresses undetected, potentially triggering or exacerbating others. High levels of stress, combined with unhealthy behaviours such as smoking or physical inactivity, significantly increase the risk of death, particularly among individuals with low socioeconomic status. This indicates that stress, unhealthy behaviours, and low socioeconomic status can collectively create a highly disadvantaged segment of the population with increased health risks [30].

Women are more likely to fall into the moderate and severe multimorbidity risk categories, pointing to gender-specific variations in life course trajectories of health. This finding is supported by studies conducted in different settings [25,31]. Studies conducted in Jamaica and the broader Caribbean region [26,32–34] have also found that women are more likely to be affected by various NCDs, which is why they also bear a higher burden of having multiple chronic conditions. Fluctuations in estrogenic and other sex hormones across the lifespan can influence the development and course of certain chronic diseases, contributing to clustering patterns. For example, highlighting the gender disparity, women are at higher risk for autoimmune diseases and osteoporosis than men [35,36]. Research has shown that sex differences in immune responses exist, influenced by various factors, including hormonal changes, genetic differences, and epigenetic adaptations. These differences can affect the development and function of the immune system, potentially leading to varied susceptibilities to diseases between males and females [37]. Epigenetics in explaining gender-specific differences in disease susceptibility has been increasingly recognised. This includes the impact of sex hormones, cellular mosaicism, and X chromosome inactivation on the immune responses of males and females, leading to different disease outcomes and susceptibilities [38]. The United Nations (UN) projected that the number of Indians over 60 would double by 2050, constituting almost 19.6% of the population [39]. To enhance health care and social service planning and improve the health and quality of life for the elderly in India, it would be beneficial to study the burden and socio-economic distribution of multi-morbidity in this population.

The frequent co-occurrence of angina, diabetes, hypertension, and stroke, often termed the ‘deadly quartet,’ suggests potential shared pathophysiological mechanisms beyond traditional risk factors [40,41]. Insulin resistance is associated with a cluster of metabolic abnormalities, including dyslipidaemia (abnormal cholesterol levels), which contributes to the development of cardiovascular diseases. The research suggests insulin resistance might lead to endothelial dysfunction and atherosclerosis, especially in diabetic patients. Endothelial dysfunction refers to the impaired functioning of the inner lining of blood vessels, which is a precursor to atherosclerosis, a condition characterised by the build-up of plaques in arteries [40,41]. Common chronic inflammatory pathways and immune responses drive the potential shared pathophysiology in asthma and arthritis. A study highlighted the role of the interleukin-17 cytokine in both conditions, indicating shared immunological mechanisms [42]. Vision impairment can significantly impact daily activities, leading to social isolation and decreased quality of life. This can contribute to the development of depression [43]. One study found a significant correlation between depression and asthma exacerbations [44]. Additionally, lung diseases, including chronic obstructive pulmonary disease, have been linked with both depression and asthma [45].

Most individuals in the moderate and severe multimorbidity risk categories point towards long-term health care facilities, highlighting the potential for improved health care systems to accommodate those needing care. The higher prevalence of asthma in moderate multimorbidity risk groups highlights the necessity for smoking prevention and cessation initiatives that specifically target this demographic. Poor environmental conditions, such as air pollution, are a significant indicator of multiple health conditions in low- and middle-income countries [46]. Arthritis, highly prevalent in the moderate multimorbidity class, generally has a multi-factorial aetiology and is a product of systemic and local factors [47]. Since the number of individuals with arthritis is likely to increase shortly due to population ageing, it will greatly impact health care and public health systems. The preponderance of angina, diabetes, hypertension, and stroke in the severe multimorbidity risk group supports the idea that there could be common physiological mechanisms involved, where the existence of one condition raises the likelihood of another, either due to shared environmental or biological risk factors [48].

In keeping with previous literature, one study found that increased waist and hip circumference also exacerbated the risk of falling into moderate and severe multimorbidity risk categories [49]. The impeccable association between an unhealthy eating pattern, obesity, being overweight, and morbidity is widely recognised [50,51]. Different modifiable lifestyle factors, such as smoking, alcohol intake, consumption of fruits and vegetables, physical activity, and risk factors like obesity, have all been linked to multimorbidity [52]. A lack of physical activity significantly increases the risk of heart disease, stroke, and high blood pressure [53]. Additionally, physical inactivity has been linked to mental health issues such as depression, anxiety, and impaired cognitive function [54]. Furthermore, obesity and high cholesterol have been shown to impact insulin sensitivity [55] and increase the risk of cardiovascular disease. Poor dietary choices and high consumption of processed foods, saturated fats, and refined sugars have been linked to chronic low-grade inflammation.

The clustering of diseases, as shown by latent class analysis, highlights the importance of integrated disease management in primary care settings. Clinicians, decision-makers, and researchers must prioritise the requirements and care procedures for patients living with or at risk of multimorbidity based on the clustering of multimorbidity and lifestyle risk factors. Patients diagnosed with a disease in primary care may be periodically tested for additional chronic illnesses using the identified disease clusters. Increased access to high-quality health care, particularly in primary health care, where many people with multimorbidity go years without receiving a diagnosis and most of those receiving treatment frequently experience uncontrolled disease.

As advocated by Barnett and colleagues, managing the clustering of diseases involves multidisciplinary teams that can provide comprehensive care, covering all aspects of a patient’s health – physical, mental, and social [56]. Regular screening for mental health issues in patients with chronic respiratory diseases or vision impairment can lead to earlier interventions, improving outcomes [55]. Patient engagement in self-care will lead to better management of chronic conditions [57]. Telemedicine and digital health platforms can significantly improve patient adherence to treatment plans and overall management of their conditions [58]. Cognitive-behavioural therapy and other forms of psychological support can help manage the mental health aspects of chronic disease [59].

In this study we looked at latent classes of a wide range of chronic illnesses in a large, nationwide sample with a representative age distribution, using a relatively recent and reasonable approach to identify the multimorbidity pattern. But some restrictions have to be cleared. A longitudinal analysis over a long period is required to estimate the incidence of transitions between latent classes and to identify characteristics associated with the development of multimorbidity of increasing severity. This is because the cross-sectional nature of the data used precludes drawing any conclusions about the temporality or causation between the chronic diseases under investigation. Further, some self-reported ailments served as the study’s foundation. Therefore, if clinical data or information about other chronic conditions were available, the multimorbidity patterns may have changed. The examined diseases might have the potential to be fatal or restrict daily activities and were included in the SAGE study. However, participants could have other chronic diseases that were not included in the list. We suggest that future studies encompass a broader range of chronic diseases to improve overall relevance. Self-reported data are inherently subjective. Terms like ‘frequent headaches’ or ‘moderate shortness of breath’ can hold vastly different meanings for individuals. This subjectivity introduces noise into the research, making comparing experiences across a diverse population challenging. Recent or significant health events tend to be etched more vividly in memory, leading to overreporting of their frequency or severity. Though there are restrictions, the results of this study strongly support the importance of examining multimorbidity patterns with latent class analysis. Moreover, replicating these results in another sample further boosts confidence in their applicability.

## CONCLUSIONS

Since understanding multimorbidity can help develop more efficient treatment and prevention strategies, in this study we revealed different population segments with distinct disease patterns. Detecting emerging pathophysiological patterns in older adults as they accumulate multiple health conditions can be crucial for preventing and managing multimorbidity. Identifying these patterns early is essential to intervene effectively and address the underlying factors contributing to developing other diseases. By recognising and addressing these patterns, health care professionals can implement preventive measures and tailor interventions to help reduce the risk of another disease in older adults. Policies promoting personalised medicine, leveraging genetic and other predictive markers, can enable prevention strategies and treatments considering individual patient complexities. Addressing social determinants through income support, affordable housing initiatives, and community-based prevention programs can mitigate their impact on health outcomes. Telemedicine, wearables, and patient portals can empower individuals to actively manage their conditions, improve medication adherence, and facilitate communication with health care providers. Policy support for digital health integration into health care systems can enhance care delivery for multimorbid patients. It is necessary to customise treatment plans for patients belonging to different multimorbidity categories, to strengthen the health and wellness centres, strengthen and deliver comprehensive primary health care services for the entire population, and to implement the Pradhan Mantri Jan Arogya Yojana for secondary and tertiary level hospitalisation services. Dealing with multimorbidity poses a challenge in managing the treatment burden for individuals with multiple diseases. The current health care system, which is fragmented and specialised, needs to incorporate and strengthen prevention and public health strategies following the needs of patients from different disease segments. Furthermore, the multimorbid segments generally have less favourable socio-demographic characteristics than the relatively healthy group. Therefore, effective and high-quality care for multimorbid patients should integrate health and social care beyond individual silos.

Utilising big data and advanced analytics to identify high-risk groups for specific multimorbidity patterns can enable targeted prevention strategies and early intervention, potentially reducing disease burden and health care costs. Research must further explore the complex interplay between social factors like stress, social isolation, and health behaviours and their role in multimorbidity development and progression. This understanding can inform interventions that address the psychosocial aspects alongside medical management. Evaluating the economic impact of different policy interventions and treatment strategies for multimorbidity is crucial for informing resource allocation and ensuring sustainable health care systems.

## Additional material


Online Supplementary Document.
