# Supplementary material for: Latent class analysis of chronic disease co-occurrence, clustering and their determinants in India using Study on global AGEing and adult health (SAGE) India Wave-2
Source: J Glob Health. 2024 Jun 28;14:04079. doi: 10.7189/jogh.14.04079 (PMC11212113; doi:10.7189/jogh.14.04079)
Supplement: Online Supplementary Document. [file jogh-14-04079-s001.pdf]

Table 1S. Latent class analysis fit statistics

Number of latent classes	Number of observations	LL	DF	AIC	BIC
Two class	6,298	-16579.8	38	33235.64	33492.06
**Three class**	**6,298**	**-16336.7**	**67**	**32807.37**	**33259.49**
Four class	6,298	-16372.5	94	32933.01	33567.32
Five class	6,298	-16290	117	32814.07	33603.58
Six class	6,298	-16227.8	153	32761.59	33794.04
Seven class	6,298	-16069.5	180	32499	33713.64
Eight class	6,298	-16060.2	206	32532.37	33922.46
Nine class	6,298	-16037.7	237	32549.45	34148.73

AIC-Akaike Information Criterion, BIC-Bayesian Information Criterion, DF-degrees of freedoms, LL-Log Likelihood

Figure S1. Latent class analysis membership of morbidities.

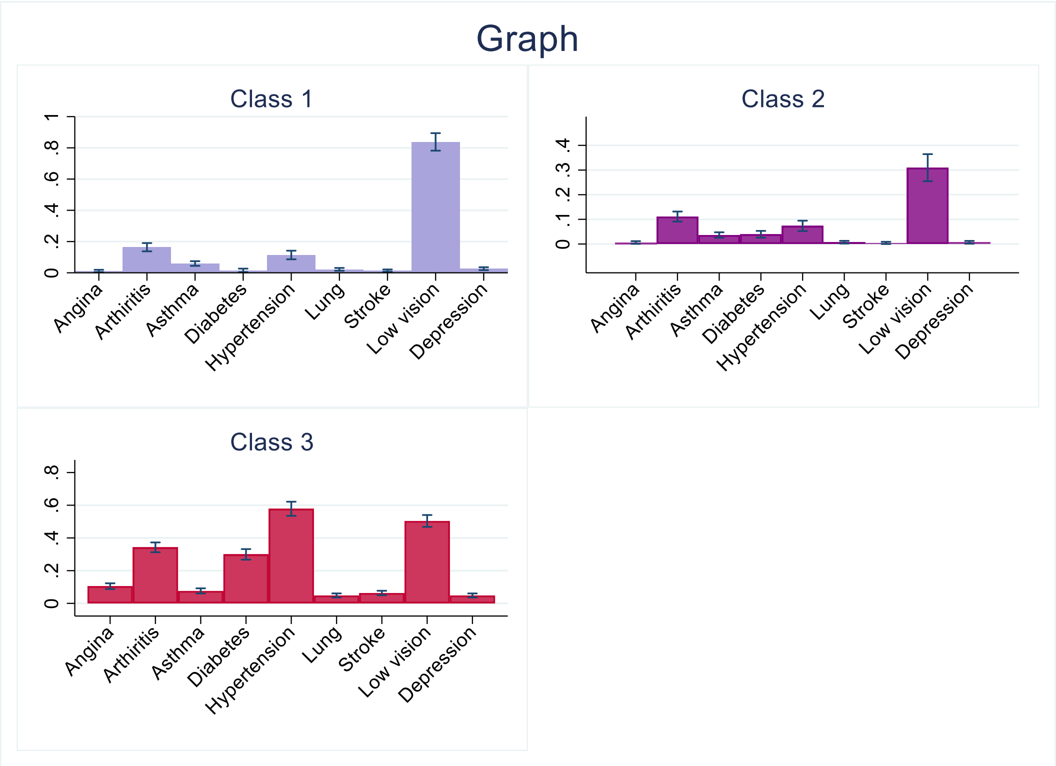

Variable categorization : The variable ‘ever worked’ indicates whether the respondent has ever worked (categorised as yes or no). Physical activity was categorised as vigorous, moderate, light, or no activity. For self-rated health, the respondents were asked how they would rate their health in general, and the response was categorised as good if they reported (good or very good), moderate, and bad if they reported their health as bad or very bad. The respondents were asked, ‘Overall, in the last 30 days, how much of a problem did you have with sleeping, such

as falling asleep, waking up frequently during the night, or waking up too early in the morning?' and their response was recorded as none, mild, moderate, severe and extreme or can't do. The sleep quality was coded as good when the response was non-mild and bad if the response was moderate, severe, extreme, or if they couldn't sleep.

Adult health-related physical activity was divided into four categories – vigorous, moderate, light, and no physical inactivity. Individuals who engage in vigorous exercise spend at least 75 minutes per week engaging in activities that cause significant increases in breathing or heart rate, such as heavy lifting, digging, or chopping wood. Individuals who engage in moderately intense exercise that causes small increases in breathing or heart rate, such as brisk walking, carrying light loads, cleaning, cooking, or washing clothes, for at least 150 minutes each week are considered to be engaged in moderate activity. Individuals who engage in a walk or use a bicycle (pedal cycle) for at least 150 minutes a week are considered to have light physical activity, and no involvement in any of the above categories is considered no activity.
